# Detecting Structural Changes in the Choroidal Layer of the Eye in Neurodegenerative Disease Patients through Optical Coherence Tomography Image Processing

**DOI:** 10.3390/biomedicines11112986

**Published:** 2023-11-07

**Authors:** Sofia Otin, Francisco J. Ávila, Victor Mallen, Elena Garcia-Martin

**Affiliations:** 1 Department of Applied Physics, University of Zaragoza, 50009 Zaragoza, Spain; avila@unizar.es; 2 Department of Ophthalmology, Miguel Servet University Hospital, 50009 Zaragoza, Spain; victormallen6@gmail.com (V.M.); egmvivax@yahoo.es (E.G.-M.); 3 Miguel Servet Ophthalmology Research Group (GIMSO), Aragon Health Research Institute (IIS Aragon), University of Zaragoza, 50009 Zaragoza, Spain

**Keywords:** artificial intelligence, choroid, neurodegenerative disease, Parkinson disease, multiple sclerosis, OCT, image processing, optical density

## Abstract

Purpose: To evaluate alterations of the choroid in patients with a neurodegenerative disease versus healthy controls, a custom algorithm based on superpixel segmentation was used. Design: A cross-sectional study was conducted on data obtained in a previous cohort study. Subjects: Swept-source optical coherence tomography (OCT) B-scan images obtained using a Triton (Topcon, Japan) device were compiled according to current OSCAR IB and APOSTEL OCT image quality criteria. Images were included from three cohorts: multiple sclerosis (MS) patients, Parkinson disease (PD) patients, and healthy subjects. Only patients with early-stage MS and PD were included. Methods: In total, 104 OCT B-scan images were processed using a custom superpixel segmentation (SpS) algorithm to detect boundary limits in the choroidal layer and the optical properties of the image. The algorithm groups pixels with similar structural properties to generate clusters with similar meaningful properties. Main outcomes: SpS selects and groups the superpixels in a segmented choroidal area, computing the choroidal optical image density (COID), measured as the standard mean gray level, and the total choroidal area (CA), measured as px^2^. Results: The CA and choroidal density (CD) were significantly reduced in the two neurodegenerative disease groups (higher in PD than in MS) versus the healthy subjects (*p* < 0.001); choroidal area was also significantly reduced in the MS group versus the healthy subjects. The COID increased significantly in the PD patients versus the MS patients and in the MS patients versus the healthy controls (*p* < 0.001). Conclusions: The SpS algorithm detected choroidal tissue boundary limits and differences optical density in MS and PD patients versus healthy controls. The application of the SpS algorithm to OCT images potentially acts as a non-invasive biomarker for the early diagnosis of MS and PD.

## 1. Introduction

Ocular medium transparency makes it possible to develop new, non-invasive techniques for diagnosing and monitoring neurological, vascular, and inflammatory pathologies. It allows practitioners to observe blood perfusion; to observe vascular wall integrity in hypertension, diabetes, and other diseases; to observe ocular or systemic diseases; and to observe axonal damage to the optic nerve in neurodegenerative disease. The eye is a window to the body and provides practitioners with non-invasive access to the brain, allowing them to study visual response and to observe the structure of the ON and retinal neurons and their distribution and characteristics [1,2,3].

Optical coherence tomography (OCT) is a technique used to diagnose ocular pathologies and to observe the retina’s neuronal and vascular structures. OCT is based on interferometry between two beams of light: a reference beam and a beam that traverses the ocular media and is refracted and reflected in their structures according to changes in their refractive indexes. Based on this information, the OCT software creates a 3D representation of the retina, allowing practitioners to observe its structure in real time. The OCT algorithm interprets the recorded interference to determine the geometric path travelled by the examination beam and expresses it as the thickness of the retinal layers. The path and interactions of the light depend on the characteristics of the medium through which it propagates, and the signal collected via OCT is influenced by this phenomenon. Ocular structures are affected in different pathologies, and the retinal changes are detectable via OCT: the OCT software estimates if there is an alteration in each eye when comparing the records against a normalized database of healthy subjects of the same age.

Many research groups have devoted their efforts to discovering how neurodegenerative diseases and the associated neuronal affectation are reflected in the optic nerve and retinal nerve fiber layer (RNFL) when evaluated via the OCT technique. Commercial OCT image processing focuses on the diagnosis of ocular disease; however, researchers use OCT to monitor and evaluate neurodegenerative disease, its progression, and even the effectiveness of treatment. Optic nerve atrophy and RNFL thinning are two findings associated with multiple sclerosis (MS). Affectation is related to the degree of severity of the disease and to the number of years since its onset. Axonal loss is generally considered to be the main disability in MS patients, and it is observable in the RNFL. Retinal macular thinning and RNFL alterations in the eyes of patients with Parkinson disease (PD) and Alzheimer disease (AD) have been detected by different research groups, and this axonal affectation has been associated with neurological progression [4,5,6]. Alterations to perivascular spaces are a potential biomarker of AD that can be observed in the retina using OCT and/or OCT-mediated angiography [7].

New OCT acquisition systems have light sources with wavelengths capable of penetrating deeper tissues and deliver high-resolution images. Subsequent software analyses of the retinal layers and choroid strengthen these findings in the study of neurodegenerative disease. In recent years, the principal aim has been to find a biomarker of neurodegenerative disease in the neuroretina or choroidal vascular tissue and to develop artificial intelligence (AI) algorithms with which to analyze images and predict or detect these pathologies [8,9,10]. These algorithms can be developed using raw data from OCT or from OCT images. Uppugunduri et al. developed an automated methodology to detect the separation of the Haller and Sattler layers of the choroid, that is, separation between the external area of large-caliber vessels versus the internal area of small-caliber vessels, with the aim of quantifying the Haller layer in the choroid using OCT images. The algorithm extracts the binarized choroidal layer, identifies the choroidal vessel, and extracts large-vessel OCT B-scan images [11]. An analysis of the choroidal tissues of MS and AD patients using OCT software showed thinning versus healthy subjects [12,13]. However, the same analysis in the eyes of PD patients shows varying results. Garcia et al. (2017) [12] detected choroid thickening in the peripapillary zone in PD patients’ eyes versus healthy eyes after comparing parameters using SS-OCT software outputs and the same findings in macular zone thickness also using SS-OCT outputs. However, in 2022, the authors used OCT-mediated angiography and did not find any differences in vasculature dynamics or structure between groups. On the other hand, Eraslan et al. (2016) and Moschos et al. (2017) found a decrease in choroidal thickness. In their work, Eraslan et al. already included image-processing procedures independent of the OCT software; they used an image-processing technique to flatten the B-scan view offered via OCT and manually delimited the distances at different pre-established points between the Bruch membrane (BM) and the hyper-reflective sclerochoroidal junction [14,15]. The aim of this study is to analyze OCT B-scan images taken from healthy subjects and patients with neurodegenerative disease using a new supervised, superpixel-based algorithm. This image-processing procedure has already shown its ability to detect and identify ellipse fitting in optic nerve fundus images from patients with glaucoma and healthy subjects with the same skill as an expert [16]. The analysis focuses on marking off boundaries in the choroidal tissue and detecting structural alterations to the choroid due to neurodegeneration. This new technique can detect gradual changes in optical density in OCT images. Our primary interest is to improve knowledge in this area, especially in patients with PD. The technique could also provide new strategies for the detection and monitoring of neurodegenerative diseases, thereby avoiding the use of more invasive techniques.

## 2. Material and Methods

### 2.1. Clinical Procedure

OCT B-scan images from a cohort comprising patients with neurodegenerative disease and healthy subjects were compiled for this study. These OCT images and choroidal area parameters were obtained from data collected during a prospective study conducted between 2015 and 2019 at Miguel Servet University Hospital (MSUH). The prospective study protocol and procedures were approved by the Aragon Research Ethics Committee (CEICA). Before their inclusion in the study, participants were required to sign an informed consent form accepting the use of pseudo-anonymized data in future research studies. In all cases, participant inclusion and data collection, storage, and processing complied with the relevant data protection legislation (Spanish Law 15/1999 on the protection of personal data, Spanish Law 14/2007 on biomedical research, and European Regulation 2016/679 on the processing of personal data). Neurodegenerative disease cohorts were recruited from the Neurology Outpatient Clinic at MSUH. The PD patients were required to meet the criteria established by the Parkinson’s UK Brain Bank [17]. The MS cohort comprised patients diagnosed with MS under standard clinical and neuroimaging criteria [18]. The participants in the healthy control cohort were spouses or relatives of the patients receiving routine care in the Ophthalmology Outpatient Clinic at MSUH. 

Before their inclusion in the study, all subjects were evaluated by means of clinical interviews and a complete ophthalmological examination to rule out the following systemic and ophthalmological alterations that could influence the OCT measurements (as seen in other studies): high refractive defects, opacity in ocular media, prior intraocular surgery, high intraocular pressure (IOP) or glaucoma, optic neuropathies, diabetes mellitus, or any cause of visual loss not attributable to the diseases of interest (PD or MS). In the prospective study, retinal OCT images were recorded by an experienced technician in the morning, using swept-source (SS) OCT technology and the HD-line examination protocol, with the Tru-Track eye-tracking technology available with the Deep Range Imaging (DRI) Triton (Topcon Corporation, Tokyo, Japan) SS-OCT device.

**Figure 1:**
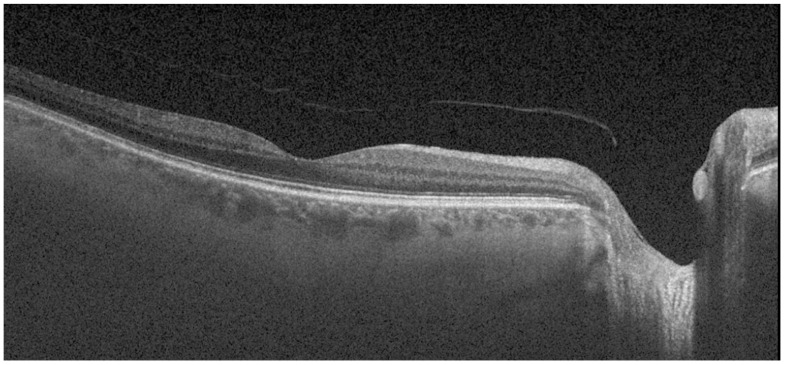
B-scan image obtained using a spectral domain optical coherence tomography (SS-OCT) device (Triton).

An independent ophthalmologist (O1) participated in the first phase of the study described below. He selected and exported high-quality OCT images according to the proposed extension to the OSCAR IB and APOSTEL guidelines for OCT images and artificial intelligence (AI) and analyzed the following data [19]. In total, 30 OCT B-scan images centered on the fovea and taken of MS patients were selected, with a gender ratio of 15:15 (female/male), an eye ratio of 14:16 (right/left), and a mean age of 61.95 (±10.09); 27 OCT B-scan images were taken of PD patients, with a gender ratio of 14:13, an eye ratio of 13:14, and a mean age of 70.72 (±6.20); and 47 OCT B-scan images were taken of age-matched healthy subjects, with a gender ratio of 23:24, an eye ratio of 22:25, and a mean age of 67.57 (±8.13). Gender, age, and intraocular pressure were considered potential confounding factors in the study. Therefore, before statistical analyses were performed, it was verified that there were no significant differences in these variables between the study groups. All groups presented *p* > 0.05. Ophthalmologist O1 collected and exported the central choroidal thickness (CCT) parameter and OCT B-scan images of one eye per patient from the above-mentioned cohorts. The images were exported in TIFF format at a resolution of 1024 × 992 pixels, as shown in Figure 1, and were randomly numbered to ensure that the other ophthalmologist (O2) was blind to the group (pathological or healthy). 

### 2.2. Image-Processing Algorithm

**Figure 2:**
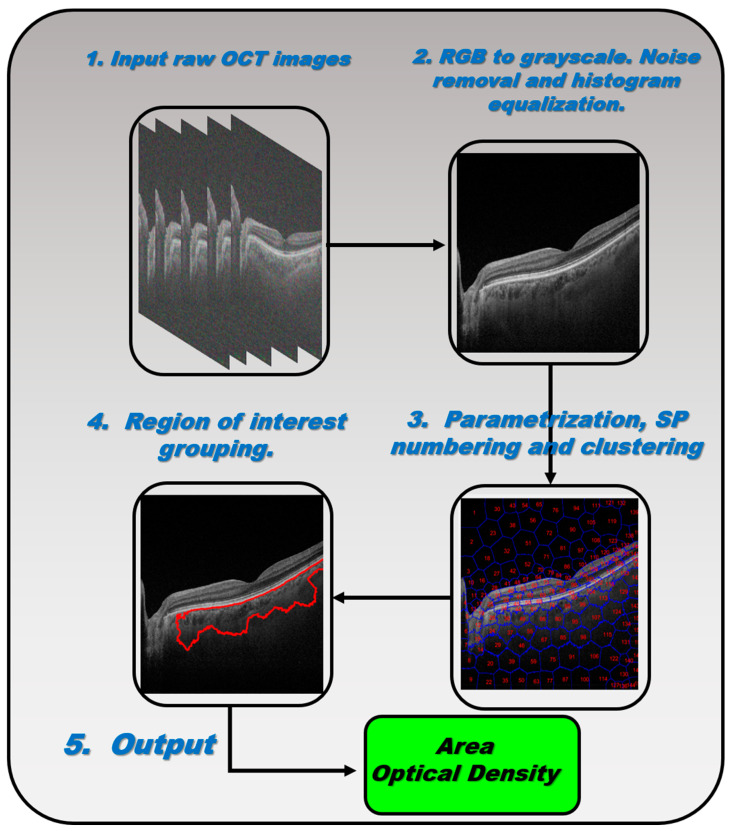
Schematics of the superpixel algorithm procedure.

Superpixel segmentation imaging [20] was employed as the main kernel to process the OCT retinal images. Briefly, the superpixel technique groups pixels with similar structural properties to generate clusters with similar meaningful properties. A custom script was written in Matlab^TM^ to execute five supervised, semi-automatic steps, as shown in Figure 2. 

Step #1: The 24-bit RGB .tiff images (1024 × 992 pixels) exported by O1 from the OCT device were converted into an 8-bit .tiff format. The converted images were cropped to form a square (992 × 992 pixels) to allow for the creation of a mathematical matrix during image processing if required. The raw images from the OCT device were saved in the working directory. Step #2: Gaussian low-pass filtering and the frequency-domain median filter were applied to remove speckle noise and power frequency artifacts, respectively, from the raw images converted in Step #1. The image contrast was then adjusted via histogram equalization to provide as flat an image histogram as possible. Step #3: Once the contrast enhancement and noise filtering were applied to the OCT images, the main function of the script called the “superpixels” Matlab function in which the number of superpixels, the number of iterations, and the regularity of the shape of the detected superpixels were parameters predefined by O2 and set for all the analyzed healthy and pathological groups. Next, the execution of the algorithm showed the numbered computed superpixels under the predefined conditions described above. At this point, O2 supervised the region of interest to be clustered by selecting those numbered superpixels that would be grouped into a single segmented retinal region, as described in Step #4. Step #4: The algorithm automatically clustered the retinal structure selected by O2 by calling the “*regionpropos*” Matlab function, which is then corrected by a supervised expert ophthalmologist to provide the optimal segmentation of the choroidal layer. Step #5: Finally, the clustered region of interest (i.e., the choroid), the optical density automatically computed by the script (measured as the standard mean gray level), and the total area of the supervised choroidal clusters were computed.

Based on the computed outputs, three parameters were defined to quantify the structural changes to the choroid due to neurodegeneration: the choroidal area (CA), defined as the total area corresponding to the segmented choroidal region of interest (pixel^2^, px^2^), the choroidal optical image density (COID), defined as the mean pixel value of the segmented area, and the choroidal density (CD), calculated as the ratio between the CA and the COID, that is, the optical density of the image per unit area.

### 2.3. Statistical Analysis

The statistical analysis was performed using IBM SPSS V.20 statistical software. Normal data distribution was detected using the Shapiro–Wilk test, and parametric tests were used for the data analysis. A one-way ANOVA was used for multiple comparisons, and a post hoc analysis was used to identify statistical differences between the groups. There was considered a statistically significant difference if the *p*-value was less than 0.05. 

## 3. Results

**Figure 3:**
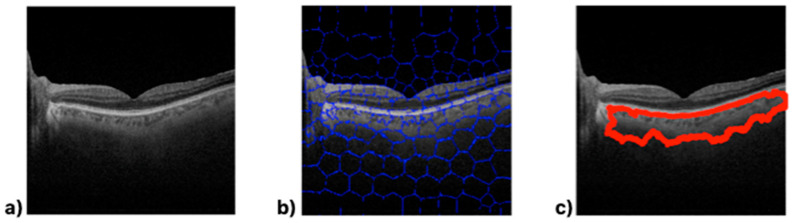
Matlab-processed image output. (**a**) Optical coherence tomography (OCT) B-scan image. (**b**) Superpixel segmentation processing result. (**c**) Choroid boundaries.

A total of 104 OCT B-scan images were processed, and the choroidal tissue was identified and delimited using the superpixel segmentation (SpS) algorithm. Figure 3 shows the choroid boundaries defined by the algorithm in an OCT B-scan image, making the differences in optical density between the different retinal layers clearly visible. 

The image obtained via algorithmic processing (Figure 3c) shows irregularities along the lower boundary. SpS detects variations in the semantic structure and delimits the area containing these changes within the image. This allows for the definition of the edges of structures; in this, case choroid boundaries. The processed images (Figure 3c) show that the cross-section selected to measure the thickness varies greatly depending on its position on the 10 mm horizontal axis because the choroid boundaries vary throughout the B-scan.

**Figure 4:**
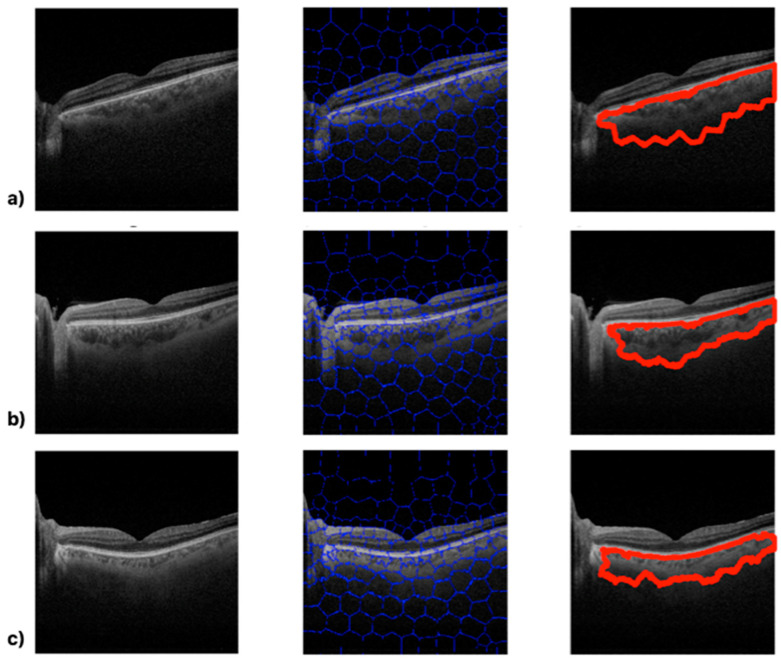
Algorithm outputs from healthy (**a**), multiple sclerosis (**b**) and Parkinson disease (**c**) subjects.

The following figures show the image processing output for three subjects chosen at random in each of the groups. The upper image (Figure 4a) shows the algorithm’s output for a healthy subject, while the middle and lower images show the algorithm’s output for MS (Figure 4b) and PD (Figure 4c) patients, respectively.

While there are no significant visually perceptible differences in the B-scan images (left-hand images in Figure 4), processing via the SpS algorithm makes the boundaries of the choroid evident. It is possible to visually detect differences between Figure 4a (a healthy eye) and the other images (pathological eyes, Figure 4b,c). The algorithm parameters mentioned above also identify clear differences according to subject type in these cases: the healthy eye (Figure 4a) presents CA = 154,940 px^2^, COID = 51.22 standard mean pixel gray level, and CD = 3025.15; the MS eye (Figure 4b) presents CA = 112,761 px^2^, COID = 62.40 standard mean pixel gray level, and CD = 1807.18; and the PD eye (Figure 4c) presents CA = 82,072 px^2^, COID = 70.95 standard mean pixel gray level, and CD = 1358.22.

**Figure 5:**
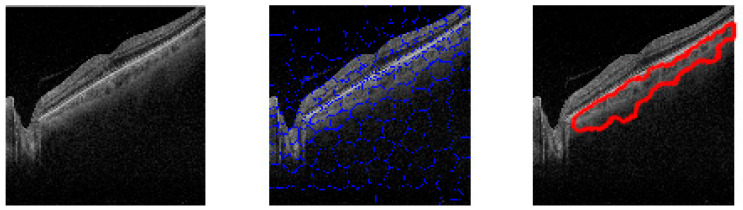
Algorithm output from a subject with Parkinson disease with a tilted retina. Original OCT b-scan left picture, clustered OCT b-scan image middle picture and choroidal area boundaries right picture.

The next image (Figure 5) shows SpS processing on a B-scan image from a tilted retina. As can be seen, the algorithm is not affected by the OCT B-scan image’s positioning issues due to the shape of the retina during the examination. 

**Figure 6:**
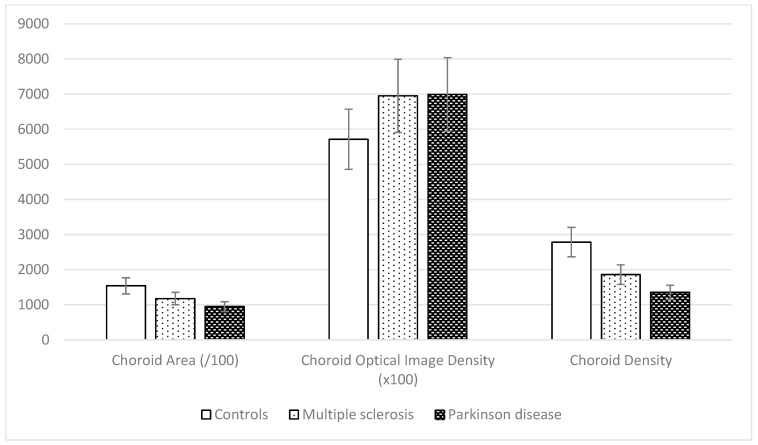
Representation with bar graph of the means and standard deviations of the analyzed parameters in the three groups: controls, multiple sclerosis, and Parkinson disease.

The algorithmic choroid analysis produced three calculated parameters. These results were analyzed according to the original group to which they belonged. The next image (Figure 6) shows a summary of the results obtained for each group, represented by the mean and the standard deviation.

The CA is greatly decreased in neurodegenerative disease patients versus healthy subjects (*p* < 0.001). The COID is higher in the neurodegenerative disease group than in the control group (*p* < 0.001), and the CD is lower in the neurodegenerative disease cohort than in the control group (*p* < 0.001). A one-way ANOVA shows statistically significant differences between groups. Table 1 shows the mean and the standard deviation in each group and the post hoc analysis differences in significance. All parameters analyzed except for the COID showed significant differences between the MS and PD groups. Similarly, the central choroid thickness and the average thickness of the choroidal layer in the sub-macular zone, calculated and delivered via OCT software, showed differences between neurodegenerative disease patients and control group subjects (*p* < 0.001). In our cohorts, the CCT is decreased in both PD and MS disease patients (246.32 ± 78.99 μm and 276.41 ± 86.10 μm, respectively) compared with healthy subjects (289.85 ± 100.14 μm).

## 4. Discussion

In recent years, research groups have focused on developing new early diagnosis and monitoring strategies for neurodegenerative disease based on objective biomarkers. They have especially concentrated on alternative, non-invasive techniques that are less expensive, safer, and more comfortable for patients than lumbar puncture to remove cerebrospinal fluid or MRI with intravenous contrast. OCT has demonstrated that it can detect neuronal alterations in retinal tissue in MS, PD, and AD patients’ eyes. These defects are considered disease biomarkers and are directly related to the degree of disease severity, the number of years since the onset of disease, and the severity scales used by neurologists to monitor neurodegenerative disease. Optical image-based diagnosis techniques have evolved, and the time taken to perform the tests and the diagnostic sensitivity of the procedures have improved. Researchers have created algorithms that facilitate clinicians’ work by processing these large masses of raw data and delivering diagnoses [8,21,22]. Artificial intelligence and the associated algorithms are capable of detecting minuscule alterations that make it possible to classify a subject as healthy or pathological. The advances in diagnostic processing techniques mean that only raw image information is required, making the procedure independent of image quality and uninfluenced by external suggestions. Thus, although poor image quality affects examiners’ decisions, the algorithm’s decisions remain constant [23].

This paper strives to advance the research group’s discoveries and developments in relation to retinal biomarkers of neurodegenerative disease, implementing these new image-processing techniques independent of the examiner and advancing the body of knowledge on the diagnosis of these diseases. Recent studies have sought to identify possible vascular changes in neurodegenerative disease. A new algorithm based on superpixel segmentation image processing was developed to study retinal vascular structures from OCT B-scan images regardless of the instrument employed. This algorithm can be used with any B-scan image generated by any commercial OCT device. In this study, only OCT B-scan images obtained using a Triton^®^ SS-OCT were used. A custom algorithm was then applied to detect retinal vascular tissue, specifically choroidal tissue, and identify possible differences between healthy subjects and neurodegenerative disease patients. Cohort members were conscientiously selected to avoid bias caused by potential confounding factors: gender, age, eye ratio, and IOP. Furthermore, only B-scan examinations performed in the morning were included to avoid possible changes associated with the circadian rhythm [24].

The results show that the processing algorithm is able to detect tissue changes in the image and can delimit the choroidal area in all subject types. An analysis of the processing images shows how these zones differ between cohorts. Based on the image data, the algorithm calculates two parameters according to the choroid image characteristics, presenting significant differences between study groups. To gain a better understanding of the COID, we can draw a comparison with X-ray imaging. Tissues vary in their density, offering varying resistance to the passage of light, analogous to how bones appear opaque in X-ray images. In our analysis, we capture the reconstructed image of the alterations that occur when an infrared light beam passes through different ocular media. The speed and direction of the propagation of the light change at each interface, leading to detectable modifications in the interference pattern upon its return (as it exits the eye). These changes manifest as distinct properties in each pixel of the image which might not be discernible to the human eye but are evident through analysis. We extract and categorize this information based on its semantic characteristics and scrutinize subtle variations. Consequently, we observe that this “obstruction” to light propagation differs based on the eye’s condition, whether healthy or afflicted by pathology. The SpS algorithm shows a good capacity to differentiate between Triton OCT images taken of neurodegenerative disease and healthy subject, and to differentiate between PD and MS eyes. This provides the potential for differential diagnosis between different neurodegenerative diseases.

Prior studies on choroidal tissue alterations in neurodegenerative disease patients show similar results. Uppugunduri et al. previously published an automated technique using open-source ImageJ software to process OCT B-scan images to detect the Haller and Sattler layers in choroidal tissue, and they centered their study on quantifying its thickness and volume. However, most published research used the software provided with the different commercial OCT devices to study the choroid. As in previous studies, this paper’s results show a smaller area of choroidal tissue than in healthy subjects. It was also possible to detect variations in optical density in MS patient choroids versus healthy eyes. Esen et al. (2016) and Garcia et al. (2018) analyzed choroidal thickness parameters obtained via different commercial OCT software from MS patients versus healthy subjects and detected that MS eyes presented a significant decrease versus healthy eyes, similar to the results of our current work [12,13,25]. Different hypotheses suggest that changes can be found in the vascular structure in MS: ganglion cell death in MS is reflected in a decrease in tissue volume and in metabolic demand to carry out its functions, which can translate into a decrease in blood flow and, therefore, in volume. Also, due to the inflammatory process associated with the disease, the walls of the vessels are damaged, which can manifest as a loss of tissue [26,27,28]. The results in this study show a reduction in the optical density of the images from MS patients that could be related to these physiological changes in blood flow and vessel properties in MS. It would be beneficial to have histological studies of the eyes of healthy subjects and neurodegenerative disease patients to test these hypotheses and to better understand the pathophysiological mechanisms affecting tissues, in particular vascular tissues such as the choroid. 

Optical al density in PD patient choroids varies versus healthy or MS eyes. The study of choroidal tissues in PD patients is a subject that generates significant controversy because the findings differ widely between authors. Our current results show a smaller area of choroidal tissue in PD patients than in healthy subjects and MS patients. Garcia et al. (2017) detected choroid thickening in the peripapillary zone in PD and detected the same findings in macular zone thickness. However, when using angiography mediated by OCT technology, they did not find any differences in vasculature dynamics or structure between groups [12,29,30]. This could be because the tissue structures observed via OCT in the choroid differ from the analysis of purely vascular structures and, mainly, of flux density in the deep and superficial choroidal plexus. Conversely, Eraslan et al. (2016) and Moschos et al. (2017) found a decrease in choroidal thickness in PD patients’ eyes in results obtained using SD-OCT outputs [14,15]. 

Previous studies used the automatic OCT segmentation technique that delimits choroidal thickness between the BM and the sclerochoroidal interface, These variations in findings in the studies may be because OCT measures thicknesses and distances at different points and, as can be seen in the OCT B-scan images obtained in this study and others, the choroid plexus boundary drawn does not need to have a regular border [31]. OCT requires the performance of an interference measurement at each point that it will calculate. Moreover, the process must be repeated in each A-scan along the B-scan length. Actually, OCT systems are able to perform up to 27,000 A-scans per sec under the best exam conditions when the patient’s eye does not present any movement and the patient is able to collaborate optimally throughout the procedure. In this case, if these A-scan measurements were totally real, it would take several minutes to perform each eye examination. OCT acquisition, however, takes no more than 3 s. Using a custom-built OCT system in laboratory research makes it possible to dedicate the time necessary to obtaining a precise measurement at each point (A-scan) because the analyzed sample does not move or grow tired; in vitro models, not patients, were used in the laboratory. In relation to this, consideration should be given to whether the data-processing function of the OCT software calculates the weighting of the values based on the data measured in the same way. 

Kwapong et al. (2018) analyzed microvascular dynamics via angiography mediated by OCT techniques and reported zones with decreased microvascular density in PD patients versus healthy subjects [32]. More recently, Robbins et al. (2021) published their results regarding choroidal dynamics and structure in PD based on angiography mediated via OCT images. They processed these images using open-source ImageJ software and performed choroidal tissue measurements using software tools. They defined two parameters of interest: the subfoveal choroidal thickness (SFCT), which is the linear distance between the outer border of the retinal pigment epithelium perpendicular to the hyper-reflective sclerochoroidal junction manually drawn on the image; and the choroidal area (CVI), which was calculated by dividing the luminal area by the total choroidal area, both of which were manually drawn on the image. Their findings showed a reduction in both parameters in PD eyes versus healthy eyes [33]. More recently, Zhang et al. (2022) used the Uppugunduri AI algorithm to analyze OCT and OCT-mediated angiography images taken of PD and healthy subjects’ eyes. Again, their study of choroidal dynamics, blood flow, and vessel volume showed significant differences between the two groups. Also, choroidal thickness was significantly decreased in the eyes of patients with PD [34].

The results obtained in this study using an independent algorithm show optical image changes in choroidal tissue in neurodegenerative disease patients versus healthy eyes. Firstly, it is evident that the boundaries defining the choroid delimit a smaller area (Figure 4). The calculated CA parameter is significantly lower in PD patients than in MS patients and in MS patients than in healthy eyes. However, this result is cannon be stated without a much larger and more diverse sample. Secondly, as reported by Zhang et al. (2022), an optical density analysis of the image (COID) shows significant differences in the choroidal vascular structure in neurodegenerative disease patients versus healthy eyes, and the relationship between the COID and the CA, choroidal density, is also significantly lower in PD patients than in MS patients and in MS patients than in healthy eyes. 

Pathophysiological choroidal changes in PD are believed to be associated with dopamine levels, which could affect blood perfusion [35,36]. Also, PD patients have increased cerebral small vessels, and decreased retinal dimensions have been observed under these conditions [37]. Moreover, the deposition of α-syn-GFP around retinal arterial vessels could be related to changes in the capillary plexus [32,38].

OCT devices detect interferometry between reference and measurement beams and, based on this information, calculate the distance travelled. In optics, distance is not only a geometrical measurement but an optical distance that depends on index refraction and geometrical distance. If the media through which light propagates change, events occur that change the optical distance. Various studies put forward different hypotheses regarding changes in vascular tissue, blood perfusion, vessel walls, etc., in PD patients. A significant change in the refractive index is necessary to produce a detectable change in light propagation; however, it cannot be ruled out that these variations exist and that they produce variations in the optical distance calculated via OCT in an interferometry analysis. An example would be a swollen cornea and how it alters the results obtained using optical diagnosis techniques. Also, commercial OCT devices do not usually consider changes in the direction of propagation in the path of the examination beam due to changes in the medium within the eye. This fact could also produce differences in the optical distances calculated and therefore in the thickness measurements.

For this reason, we propose not focusing on an isolated measurement of uncertain origin, such as the thickness of the choroidal structure. It is evident that the extent of the discrepancies and variability in the results is due to reasons yet unknown. The area parameter, the optical density image, and other parameters previously mentioned and obtained automatically from an OCT image are more reliable because they allow for the elimination of many of the factors listed above. It is becoming clear that changes occur in the choroidal vascular structure in PD subjects, and more studies are necessary. AI procedures can help to find these differences and define a pattern, but it is necessary to have a large number of standardized, high-quality images that can be processed. Other imaging techniques, such as MRI, have public databases containing thousands of images and the corresponding diagnoses with which to develop this type of diagnostic support algorithm based on deep learning.

The non-commercial superpixel segmentation algorithm used in this study allows for the detection of choroidal boundaries and its alterations in neurodegenerative disease by processing OCT images. This method may be used in the early diagnosis of MS or PD and has the potential to be a non-invasive, easily tolerated, low-cost, and effective tool for the early diagnosis and even differential diagnosis of neurodegenerative disease. In addition, it is an algorithm that can process images obtained using any OCT device, making it easy to integrate into clinical practice. Our calculation of the choroidal optical image density, achieved through semantic processing, is shown as the COID parameter. This demonstrates variances between the eyes of healthy individuals and those with ocular diseases, suggesting it could serve as a potential biomarker for structural changes in choroidal vascular tissue. The use of non-invasive OCT techniques for in vivo measurements provides an avenue for enhancing our understanding of vascular tissue and identifying early abnormalities. To delve deeper, further research involving patients with vascular diseases or ocular vascular pathologies is essential. We anticipate that this work will pave the way for future collaborations and the development of this promising research line.

## Figures and Tables

**Table 1:** Means ± standard deviations of the proposed parameters for each group obtained via image processing and significance of the comparison between the three groups with the post hoc analysis results.

	Control Group	MS Cohort	PD Cohort	Significance
CA	154,071.53 ± 20,948.58	117,736.13 ± 16,153.50	94,722.85 ± 19,510.99	*p* < 0.001(C vs. MS *p* < 0.001, C vs. PD *p* < 0.001, MS vs. PD *p* < 0.001)
COID	57.14 ± 6.09	64.49 ± 7.71	69.90 ± 6.74	*p* < 0.001 (C vs. MS *p* < 0.001, C vs. PD *p* < 0.001, MS vs. PD *p* = 0.013)
CD	2784.82 ± 562.93	1858.95 ± 370.34	1354.36 ± 395.77	*p* < 0.001 (C vs. MS *p* < 0.001, C vs. PD *p* < 0.001, MS vs. PD *p* = 0.001)

Measurements of the choroid area (CA) are in pixels squared (px^2^). Measurement of the choroid optical image density (COID)—standard mean pixel grey level. The choroid density (CD) is a dimensionless parameter. Abbreviations: CA—choroid area; COID—choroid optical image density; CD—choroid density; v.s.—versus; MS—multiple sclerosis; PD—Parkinson disease.

## Data Availability

The datasets are available from the corresponding author upon reasonable request.
